# Virchow on Soups and Broths

**Published:** 1882-06

**Authors:** 


### VIRCHOW ON SOUPS AND BROTHS.

This distinguished German professor and politician has been accused of being the chief opponent of soup. He says that this is not true, for he had merely said that meat broths are neither nutritious nor "substantial." That if all the meat which one uses should be boiled and soup made of it the meat would become for the greater part indigestible, and the soup would not be a substitute for it. Broth, he says, is an article of luxury which only the comparatively well-to-do can afford. A family that can only just make both ends meet should learn to deny themselves this luxury, since they have a similar one in their coffee. A

rich man can afford to eat soup; while the sick sometimes must have it.

Ordinary meat broth or bouillon in its pure form can only be recognized as a condiment. By the addition of eggs, flour, fat, and other things it may acquire a certain nourishing and heating value. It is, primarily, only a very dilute aqueous solution of substances that are in part of low value as heat producers, such as gelatine, and in part of the stimulating aromatic parts of the meat. Taken warm it is of nearly the same value as coffee or tea, but is inferior to wine, schnapps, or beer, it only stimulates the nerves. It has one advantage over every other condiment, namely, it contains no poisonous substance, it is incomparably milder, hence much better adapted to feeble persons, and finally it can be very conveniently combined with substances that are actually nutritious. and imparts to them an agreeable and "substantial" taste.

It must be admitted that these stimulants (soup and coffee), because they are stimulants, have more significance than mere condiments. By their stimulating power they awaken the slumbering energies. So long as power is left to exert this energy these stimulants are able to vitalize these forces. Hence it produces the impression of being itself strengthening. It has not of itself this power; it can only awaken other forces already present, but cannot create them. A tired organ, a tired laborer, can find new strength in a stimulant because it arouses within him certain powers which would not otherwise have come to his aid. In this lies the secret, and at the same time the beneficial effect, of many stimulants, so that they are, of course, more than mere condiments or flavors, and become, to a certain extent, tools. Used in moderation they can do much good in this direction. But it must not be forgotten that they are not food, and that every energy brought forth by stimulants requires a double influx of substance to replace that consumed, so that it may not result in exhaustion. Condiments can never take the place of nourishing food.

A large portion of our food, it is true, acts at the same time as a condiment, and even as a stimulant. By this is not meant those natural mixtures of nutritive and stimulating substances so frequently found combined in vegetables, nor yet those artificial compounds prepared by skilled cooks, but rather food which has been eaten refreshes and strengthens a person long before the real digestion has been finished. A laborer, who is tired and hungry, has set before him a meal of meat and potatoes, and as soon as his meal is eaten he feels refreshed and ready for work again. Nevertheless it is three or four hours before the meat is dissolved and absorbed into the blood, and even if a portion of the potato starch is converted into sugar or glucose while he is chewing it, it is decidedly the smallest portion. The feeling of strength which the man is sensible of cannot possibly come from the assimilation of his food into the tissues. Its direct effect upon the surface of the organs of digestion and a very slight absorption of the material into the blood exert sufficient stimulus to overcome or relieve the weary condition. It is only on this ground that we can explain why a drink of fresh cool water, a sip of wine or beer, seems to be as invigorating as, or even more so than, a piece of roast beef, although not to be compared with it in permanent effects.

The first invigorating effect that we experience after a meal is either due to the action of the condiment or is the result of those properties of food which place them on the same footing with mere condiments. Afterward the true digestion takes place, the replacing of the material consumed in work, and with it the sensation of permanent strengthening.

It is this point of view which is often lost sight of by the new school of physiologists who treat of nature and sustenance. The confusion that exists in regard to the best method of giving nourishment is a natural result of the very one-sided treatment of the whole question, from a purely chemical view, and the error is increasing rather than otherwise. Chemical investigations have a very subordinate importance in recognizing the exciting power of real food and of condiments; the physiological view must here be taken. Virchow, therefore, attempts to restore to the latter science, physiology, its old rights, and hopes to protect scientists and laity from that one-sidedness which always supplants one error by another, and which has nowhere led to more visible results than in this important and interesting domain.

The words of so careful a writer and so thorough an investigator deserve the attention of thinking men on both sides of the question.—*Scientific American.*

